# Tafasitamab mediates killing of B-cell non-Hodgkin’s lymphoma in combination with γδ T cell or allogeneic NK cell therapy

**DOI:** 10.1007/s00262-022-03165-w

**Published:** 2022-03-29

**Authors:** Jung Hyun Her, Dominik Pretscher, Maria Patra-Kneuer, Juergen Schanzer, Sung Yoo Cho, Yu Kyeong Hwang, Timm Hoeres, Rainer Boxhammer, Christina Heitmueller, Martin Wilhelm, Stefan Steidl, Jan Endell

**Affiliations:** 1 Cell Therapy Research Center, GC LabCell, Yongin, Republic of Korea; 2 Department of Hematology and Medical Oncology, Paracelsus Medical University, Nuremberg, Germany; 3 MorphoSys AG, Planegg, Germany

**Keywords:** NK cells, γδ T cells, Tafasitamab, ADCC, B-cell non-Hodgkin’s lymphoma, Immunotherapy

## Abstract

Tafasitamab is an Fc-modified monoclonal antibody that binds to CD19, a cell-surface antigen that is broadly expressed on various types of B-cell non-Hodgkin’s lymphoma (NHL). Antibody-dependent cellular cytotoxicity (ADCC), a key mode of action of tafasitamab, is mediated through the binding of tafasitamab’s Fc region to FcγRIIIa receptors on immune effector cells and results in antitumor activity. Despite the proven clinical activity of tafasitamab in combination with lenalidomide in the treatment of diffuse large B-cell lymphoma (DLBCL), a higher number of immune cells in cancer patients may improve the activity of tafasitamab. Here, we characterized two ex vivo-expanded FcγRIIIa receptor—expressing cell types—γδ T and MG4101 natural killer (NK) cells—as effector cells for tafasitamab in vitro, and found that in the presence of these cells tafasitamab was able to induce ADCC against a range of NHL cell lines and patient-derived cells. We also explored the concept of effector cell supplementation during tafasitamab treatment in vivo by coadministering MG4101 NK cells in Raji and Ramos xenograft models of NHL. Combination treatment of tafasitamab and allogeneic MG4101 NK cells in these models demonstrated a survival benefit compared with tafasitamab or MG4101 monotherapy (Raji: 1.7- to 1.9-fold increase in lifespan; Ramos: 2.0- to 4.1-fold increase in lifespan). In conclusion, adoptive cell transfer of ex vivo-expanded allogeneic NK or autologous γδ T cells in combination with tafasitamab treatment may potentially be a promising novel approach to increase the number of immune effector cells and enhance the antitumor effect of tafasitamab.

## Introduction

Tafasitamab (MOR208) is an Fc-modified, humanized monoclonal antibody that binds to the human B-cell surface antigen CD19 [1, 2]. CD19 is broadly and homogeneously expressed across B-cell malignancies, including diffuse large B-cell lymphoma (DLBCL), chronic lymphocytic leukemia (CLL), Burkitt’s lymphoma (BL), and mantle cell lymphoma (MCL) [3]. Tafasitamab’s modes of action include antibody-dependent cellular cytotoxicity (ADCC), antibody-dependent cellular phagocytosis, and direct cytotoxicity (apoptosis) [1, 2]. In vitro, tafasitamab has shown cytotoxicity against a range of B-cell lymphoma and leukemia cell lines [1, 2]. In a Phase II clinical study with relapsed or refractory (R/R) DLBCL patients, tafasitamab in combination with the immunomodulatory drug lenalidomide was well tolerated and resulted in 60% of patients achieving an objective response, with a median duration of 21.7 months [4]. Based on this study, tafasitamab in combination with lenalidomide received accelerated approval by the Food and Drug Administration in 2020 for the treatment of transplant-ineligible adult patients with R/R DLBCL.

Tafasitamab has two amino acid substitutions in the Fc region (S239D/I332E) that increase its affinity for Fcγ receptors, including FcγRIIIa (CD16) [2]. FcγRIIIa plays a key role in mediating ADCC and is expressed on the surface of natural killer (NK) cells and most γδ T cells [5]. The ADCC potency of tafasitamab in vitro is 100- to 1000-fold higher relative to an unmodified anti-CD19 IgG1 analogue [2], suggesting an important role for CD16-positive immune effector cells in ADCC-mediated antitumor activity.

Several clinical trials have reported that low numbers of NK cells in peripheral blood adversely affect the treatment outcomes of patients with lymphomas undergoing immunotherapy [6, 7]. Furthermore, γδ T cells in cancer patients have been found in reduced numbers and with a diminished proliferative capacity relative to healthy donors [8–10]. A Phase I clinical study of patients with malignant lymphoma or advanced solid tumors who were treated with healthy human donor–derived, ex vivo*-*expanded allogeneic NK cells (MG4101) reported stable disease in 8 of 17 evaluable patients (47.1%) and a promising safety profile [11]. Similarly, adoptive immune cell therapy with autologous γδ T cells demonstrated a low toxicity profile with modest therapeutic efficacy as monotherapy in several clinical trials [10]. Therefore, combination of tafasitamab with adoptive NK or γδ T cell therapy may potentially be a promising novel approach to increase the number of immune effector cells in the tumor microenvironment and enhance the antitumor effects of tafasitamab. Prior studies have shown enhanced cytotoxicity of γδ T cells or allogeneic NK cells against a variety of tumor types upon combination with therapeutic antibodies, including rituximab and trastuzumab [12–15].

To explore this concept for tafasitamab, we confirmed the ability of ex vivo-expanded γδ T or allogeneic NK cells (MG4101) to mediate tafasitamab-induced ADCC against lymphoma cells in vitro. In addition, we investigated the potential of adoptive cell therapy in combination with tafasitamab by supplementing tafasitamab treatment with MG4101 ex vivo-expanded NK cells in disseminated tumor mouse models of NHL.

## Materials and methods

### Antibodies

Tafasitamab and XmAb5603, an anti-CD19 analogue of tafasitamab with a wild-type IgG1 Fc region, were provided by MorphoSys AG. As controls, either a polyclonal IgG1 isotype control (Sigma I5154) or an IgG control (Sigma I4506) were used.

### Cell isolation and culture

#### γδ T cells

γδ T cells were generated by zoledronate and interleukin-2 stimulation and culture of peripheral blood mononuclear cells (PBMCs) from different healthy donors for 9–12 days as described previously [16].

#### MG4101 NK cells

PBMCs were isolated from healthy donors by leukapheresis. NK cells were enriched, expanded, and cryopreserved as described previously [17].

#### Primary human leukemia and lymphoma cells

Primary human leukemia and lymphoma cells were isolated from heparinized peripheral blood or bone marrow biopsies of patients recruited at the Nuremberg University Hospital, Germany.

#### Cell lines

Jeko-1, Mino, Daudi, U-2932, Raji, and Ramos cell lines were obtained from the German Collection of Microorganisms and Cell Cultures or the American Type Culture Collection and cultured in standard medium.

### Cytotoxicity assays

#### γδ T cells as effector cells

ADCC with γδ T cells was determined by flow cytometry as described previously [16]. After incubation of carboxyfluorescein succinimidyl ester (CFSE)-labeled target cells (*T*) with antibody and effector cells (*E*) for 4 h at 37 °C and *E*/*T* ratios ranging from 0.7:1 to 20:1, cells were harvested, stained with TO-PRO-3, and evaluated by flow cytometry. Cell lysis at each *E*/*T* ratio was calculated using the following formula: ${\text{Cell lysis}}\,\left( \% \right) = \frac{{\left( {\% {\text{TO-PRO-3-positive cells}} - \% {\text{TO-PRO-3-positive cells in target cell-only culture}}} \right)}}{{\left( {{100} - \% {\text{TO-PRO-3-positive cells in target cell-only culture}}} \right)}} \times {100}.$

#### Allogeneic NK cells as effector cells

For quantification of ADCC, target cells were labeled with calcein-AM. Monoclonal antibodies (tafasitamab or control IgG) were added to the target cells and incubated with MG4101 NK effector cells at an *E*/*T* ratio of 3:1. After incubation for 2 h, plates were centrifuged and calcein release was quantified using a fluorescence microplate reader. As a control, maximal lysis (release) was induced with 1% Triton *X*-100. The percentage of ADCC-mediated cell lysis was measured by calcein release from target cells and calculated using the following formula: ${\text{Cell lysis}}\; \left( \% \right) = \frac{{\left( {{\text{sample release}} - {\text{spontaneous release}}} \right)}}{{\left( {{\text{maximal release}} - {\text{spontaneous release}}} \right)}} \times {100}.$

#### In vivo lymphoma xenograft models

For the disseminated Raji model, 1 × 10^5^ Raji cells/mouse were intravenously (i.v.) injected into the tail vein of CB17/Icr-Prkdcscid/CrlCrlj (CB-17 SCID) mice on Day 0 and cryopreserved MG4101 NK cells (2 × 10^7^ cells/mouse) or freezing medium (vehicle control) were administered i.v. on Days 1, 3, 6, 8, and 10. Tafasitamab was administered subcutaneously (s.c.) once on Day 1 at a dose of 2.5 μg/kg. In the disseminated Ramos model, 1 × 10^6^ Ramos cells/mouse were injected i.v. on Day 0 and MG4101 NK cells were administered i.v. on Days 4, 7, 11, 14, 18, and 21. Tafasitamab was administered i.v. on Days 3, 6, 10, 13, 17, and 20 at a dose of 10 mg/kg. Mice were monitored daily for tumor-associated morbidity, mortality, and hind limb paraplegia.

All animal experiments were approved by the Institutional Animal Care and Use Committee of the Korea Research Institute of Bioscience & Biotechnology.

### Data and statistical analysis

Statistical significance in ADCC experiments was calculated using a two-tailed paired *t* test. Increased life span (%) in mouse models of survival was calculated using the following formula: ${\text{Increased life span}}\;\left( \% \right) = \frac{{\left( {{\text{median survival treatment}} - {\text{median survival control}}} \right)}}{{\text{median survival control }}}{ } \times {100}.$The log-rank test was used to compare survival of control and test groups. A *P *value < 0.05 was considered statistically significant for all experiments.

## Results

### Tafasitamab induced killing of NHL cell lines and primary CLL and MCL cells by γδ T cells

**Fig. 1:**
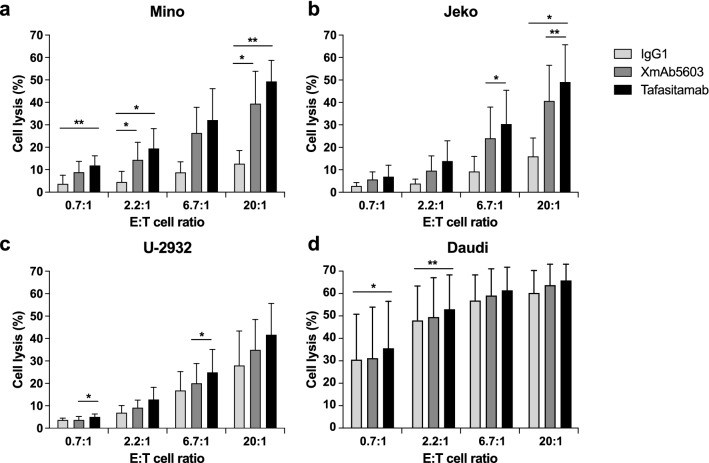
γδ T cell ADCC assays against lymphoma cell lines. Mino (**a**), Jeko (**b**), U-2932 (**c**), and Daudi (**d**) target cells (*T*) were combined with γδ *T* effector cells (*E*) at different ratios. Cells were incubated for 4 h with 1 μg/mL of tafasitamab, XmAb5603, or IgG1 isotype as a control. Cell lysis was determined by fluorescence-activated cell sorting (FACS) analysis of CFSE- and TO-PRO-3-stained target cells. Data are mean values plus standard deviation for three different assays using γδ T cells derived from three different healthy donors. *P < 0.05; **P < 0.01

ADCC assays using γδ T cells expanded from PBMCs of three healthy human donors demonstrated that tafasitamab induced cytotoxicity against Mino and Jeko (MCL) cell lines in an *E*/*T* ratio-dependent manner (Fig. 1a, b). Furthermore, part of this activity was related to tafasitamab’s Fc-enhancement because an unmodified IgG1 analogue (XmAb5603) induced a lower ADCC. Although similar levels of tafasitamab-induced cytotoxicity were observed against U-2932 (DLBCL) cells, no meaningful conclusion could be drawn for Daudi (BL) cells because of high background levels of cell death (Fig. 1c, d).

**Fig. 2:**
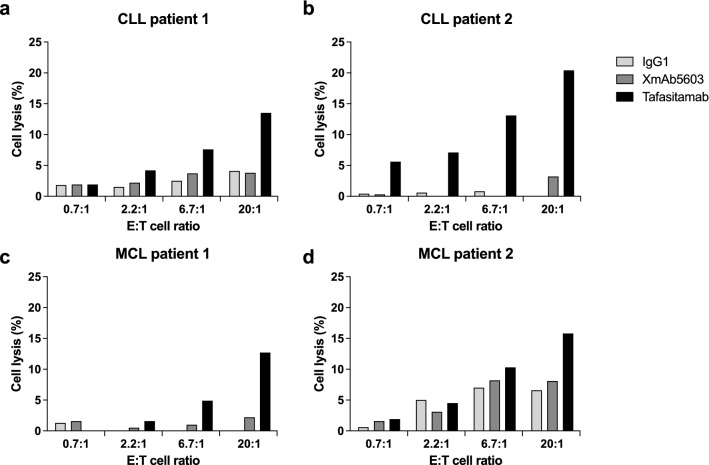
γδ T cell ADCC assays against leukemia (CLL) and lymphoma (MCL) cells from patient samples. Cells from two different patients with CLL (**a**, **b**) and MCL (**c**, **d**) were used as target cells (*T*). The target cells were combined with γδ *T* effector cells (*E*) from one healthy donor and tested at different *E*/*T* ratios. Cells were incubated for 4 h with 1 μg/mL of tafasitamab, XmAb5603, or IgG1 isotype as a control. Cell lysis was determined by FACS analysis of CFSE- and TO-PRO-3-stained target cells. Because of limited availability of patient samples, single measurements were performed for each condition

Next, we analyzed the ADCC of γδ T cells in combination with tafasitamab against primary CLL and MCL cells derived from two individual patients per indication. Whereas tafasitamab induced tumor cell death in an *E*/*T* ratio–dependent manner, XmAb5603 showed no substantial increase in cell death compared with background levels (Fig. 2). Altogether, these findings demonstrate that γδ T cells could serve as effector cells for tafasitamab by mediating ADCC against NHL cell lines and primary tumor cells.

### Combination of tafasitamab and allogeneic NK cells led to increased antitumor activity in disseminated BL mouse xenograft tumor models

**Fig. 3:**
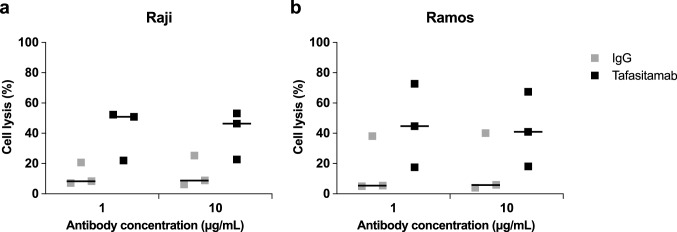
Allogeneic NK cell ADCC assays against lymphoma cell lines. The B-cell lymphoma Raji (**a**) and Ramos (**b**) cell lines were incubated for 2 h with saturating concentrations of tafasitamab or IgG isotype control (1 or 10 μg/mL) in the presence of expanded MG4101 NK cells from three healthy donors at an *E*/*T* ratio of 3:1. Calcein release from labeled target cells was measured using a fluorescence microplate reader. Results from three different donors (squares) and the median values (solid lines) are depicted

Prior to in vivo studies, the ability of ex vivo-expanded, cryopreserved MG4101 NK cells (isolated from three healthy donors) to mediate tafasitamab-induced ADCC was evaluated in vitro using Raji and Ramos cell lines. Compared with the IgG isotype control, tafasitamab induced increased ADCC against both Raji and Ramos cell lines (Fig. 3).

**Fig. 4:**
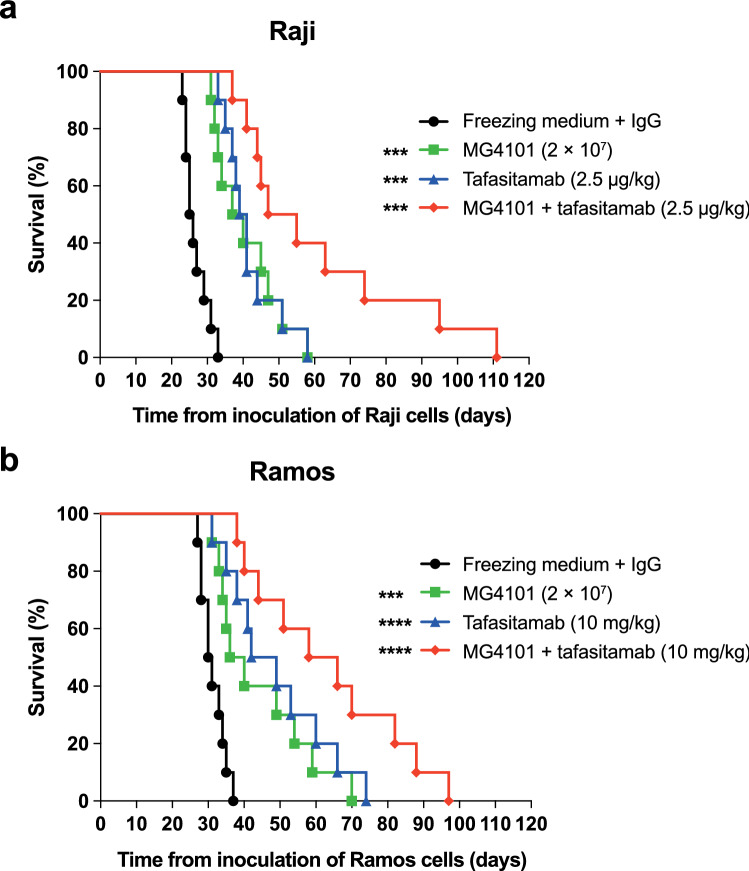
Survival of disseminated xenograft mice models treated with MG4101 NK effector cells and/or tafasitamab. For the Raji model (**a**), groups of 10 SCID mice were injected i.v. with 1 × 10^5^ Raji cells (Day 0). Mice were then injected s.c. with a single dose of tafasitamab (2.5 μg/kg) on Day 1 and/or i.v. with MG4101 (2 × 10^7^ NK cells) on Days 1, 3, 6, 8, and 10. Median survival was 25.5, 38.5, 40.0, and 51.0 days for mice treated with freezing medium + control IgG, MG4101, tafasitamab, and MG4101 + tafasitamab, respectively. For the Ramos model (**b**), groups of 10 SCID mice were injected i.v. with 1 × 10^6^ Ramos cells (Day 0). Mice were then injected i.v. with tafasitamab (10 mg/kg) on Days 3, 6, 10, 13, 17, and 20 and/or i.v. with MG4101 (2 × 10^7^ NK cells) on Days 4, 7, 11, 14, 18, and 21. Median survival was 30.5, 38.0, 45.5, and 62.0 days for mice treated with freezing medium + control IgG, MG4101, tafasitamab, and MG4101 + tafasitamab, respectively. Statistically significant differences compared with the freezing medium + control IgG are indicated. ***P < 0.001; ****P < 0.0001

Next, tafasitamab in combination with MG4101 immune cell supplementation was evaluated in two disseminated BL survival models in severe combined immunodeficiency (SCID) mice. First, in a preventive Raji model, a single subcutaneous low dose of 2.5 μg/kg tafasitamab was given on Day 1 after tumor inoculation, and MG4101 cells were administered i.v. on Days 1, 3, 6, 8, and 10. Combination treatment resulted in a greater increase in lifespan (ILS) than tafasitamab and MG4101 monotherapies (ILS 100% vs. 57% and 51%, respectively; increase in lifespan 1.7- to 1.9-fold; Fig. 4a).

Second, in a Ramos BL tumor model, SCID mice were treated i.v. with 6 doses of 10 mg/kg tafasitamab on Days 3, 6, 10, 13, 17, and 20 after tumor inoculation. In this model, increased survival was observed for the tafasitamab-treated mice compared with the IgG isotype control group (ILS 49.2%; Fig. 4b). MG4101 cell therapy was given on Days 4, 7, 11, 14, 18, and 21, leading to an ILS of 24.6%. In contrast, combination therapy (tafasitamab plus MG4101 cells) resulted in an ILS of 103.3%, which was more than additive of the ILS of each monotherapy (increase in lifespan 2.0- to 4.1-fold; Fig. 4b).

## Discussion

This study examined the suitability of ex vivo-expanded NK and γδ T cells as effector cells for tafasitamab-induced cytotoxicity against various NHL subtypes, including MCL, CLL, and BL, in vitro and in vivo. In clinical trials of immunochemotherapy-treated NHL patients, low numbers of NK cells in peripheral blood or tumor tissue prior to treatment have been linked with poor outcomes [4, 6, 7]. Therefore, we hypothesized that supplementing effector cells could potentially enhance tafasitamab’s antitumor activity.

The feasibility of such an approach has been reported recently in a Phase I clinical study of R/R B-cell NHL patients who were treated with the CD20-targeted antibody rituximab and MG4101 allogeneic NK cells [18]. Here, we explored the feasibility of using tafasitamab, a therapeutic antibody that targets a different antigen: CD19. In SCID mice carrying disseminated Ramos tumors, MG4101 plus tafasitamab combination treatment significantly increased survival compared with the respective monotherapies. A similar survival benefit was also observed when using a single low dose of tafasitamab in a preventive Raji model to simulate minimum residual disease and potentially low antibody concentrations in the tumor. These data are in line with an earlier study using a Raji tumor model where mice treated with rituximab plus NK cells showed a significant increase in survival [19].

As an alternative to adoptive NK cell therapy, we focused on γδ T cells, which are able to infiltrate different cancerous tissues and inhibit tumor growth and metastases [16]. Hoeres et al. previously demonstrated that combination of γδ T cells with the CD20-targeted antibodies rituximab and obinutuzumab resulted in elimination of MCL and CLL lymphoma cells in vitro [16]. However, as CD16 expression on γδ T cells is weaker than on NK cells, and CD19 is often expressed at lower levels on target lymphoma cells than CD20 [5, 20], it was important to confirm that these immune cells can indeed serve as effector cells for tafasitamab, especially against primary lymphoma cells. The activity of tafasitamab observed in our study was similar to the activity previously reported with CD20-targeted antibodies [16].

In vitro analyses of ex vivo-expanded NK and γδ T cells by Niu et al. showed that γδ T cells had a higher proliferative capacity than NK cells, whereas NK cells had a stronger ability to secrete cytokines and a higher overall cytotoxicity [21]. These data provide rationale that the clinical administration of either cell type in combination with tafasitamab treatment could potentially be beneficial for patients. Overall, allogeneic immune cell–antibody combination therapies may have advantages over autologous cell transfer strategies because the former have fewer manufacturing challenges and cryopreserved batches are readily available for treatment. Moreover, whereas allogeneic NK cell therapy and tafasitamab treatment have both been reported to be well tolerated as monotherapies in clinical trials [4, 22], other cellular therapies such as CAR-T cells are often associated with toxicities such as cytokine release syndrome and neurotoxicity [23].

In conclusion, we have provided preclinical evidence showing that combinations of tafasitamab with γδ T or allogeneic NK cells could be promising strategies for leveraging the full therapeutic potential of tafasitamab against CD19-positive B-cell tumors.

### About tafasitamab

Tafasitamab is a humanized Fc-modified cytolytic CD19-targeting monoclonal antibody. In 2010, MorphoSys licensed exclusive worldwide rights to develop and commercialize tafasitamab from Xencor, Inc.

Tafasitamab incorporates an XmAb® engineered Fc domain, which mediates B-cell lysis through apoptosis and immune effector mechanism including antibody-dependent cell-mediated cytotoxicity (ADCC) and antibody-dependent cellular phagocytosis (ADCP).

In January 2020, MorphoSys and Incyte entered into a collaboration and licensing agreement to further develop and commercialize tafasitamab globally. Following accelerated approval by the U.S. Food and Drug Administration in July 2020, tafasitamab is being co-commercialized by MorphoSys and Incyte in the United States. Incyte has exclusive commercialization rights outside the United States.

XmAb® is a trademark of Xencor, Inc.

## Data Availability

Data may be provided for reasonable academic studies upon request by the corresponding author.
